# Case Report: A Rare Cause of Stridor and Hoarseness in Infants—Congenital Laryngeal Web

**DOI:** 10.3389/fped.2022.875137

**Published:** 2022-04-08

**Authors:** Yanyan Wang

**Affiliations:** Department of Respirology, Children’s Hospital of Hebei Province, Shijiazhuang, China

**Keywords:** congenital laryngeal web, infant, stridor, hoarseness, bronchoscopy

## Abstract

**Background:**

Congenital laryngeal web is a rare disease that can cause stridor, dyspnea, hoarseness, and other symptoms. Children with this disease generally have stridor, weak crying, and hoarseness at birth, but these symptoms can also occur during the days, weeks, months, and even years after birth. Respiratory tract infection will obviously aggravate these manifestations, and recovery is slow after symptomatic treatment. Neck CT and laryngoscopy can detect and diagnose this disease. It is important that the disease can be considered and examined in infants with recurrent stridor and persistent hoarseness after birth.

**Case Summary:**

We report a 23-month-old boy who was admitted to hospital due to stridor and hoarseness after birth. Combined with the results of laryngoscopy, he was diagnosed with congenital laryngeal web, and was treated with carbon dioxide (CO_2_) laser and cryotherapy by bronchoscope. The prognosis was good.

**Conclusion:**

Congenital laryngeal web is a rare but challenging laryngeal lesion. It is very important that the disease can be considered and examined for infants with recurrent stridor and persistent hoarseness after birth. The treatment strategy after diagnosis should be determined according to the classification of laryngeal web and the severity of children’s symptoms.

## Introduction

The most common symptoms of infants in respiratory department are cough, stridor, hoarseness, and shortness of breath, with or without fever. They are often diagnosed as having common illnesses such as laryngitis, bronchopneumonia, or laryngomalacia. However, when the child has a medical history of recurrent stridor and hoarseness after birth, or is not satisfied with the therapeutic effects of glucocorticoid and antibiotics, unusual causes should be considered. Congenital laryngeal web is one of the rare congenital causes of stridor and hoarseness. The child’s age and medical history are critical to the diagnosis of this disease. In this paper, we present a case of a 23-month-old boy who was admitted to hospital due to stridor, weak crying, and hoarseness after birth, who was diagnosed as congenital laryngeal web and recovered well after laryngeal web separation.

## Case Presentation

**FIGURE 1:**
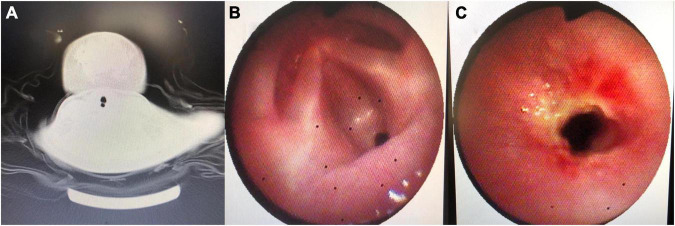
**(A)** Neck CT noting linear diaphragm in glottis. **(B)** Image from the first bronchoscopy: webbed connection covering the anterior commissure of glottis and blocking nearly two thirds of glottis. **(C)** Image from the second bronchoscopy: a small amount of yellow necrotic material attaching to the anterior commissure of glottis.

A 23-month-old boy was admitted to hospital after a 5-day history of stridor, cough, and hoarseness. On the day of admission, he was in good spirits and had clear consciousness, oxygen saturation of 96% on room air, body temperature of 36.8^°^C, breathing rate of 34/min, and heart rate of 116/min. The suprasternal fossa, supraclavicular fossa, and intercostal space were retractions during inhalation. Inspiratory stridor and moist rales could be heard in the lungs. The cardiac examination result was found to be normal. Asked about history, the boy was a full-term child born through vaginal delivery, with normal birth history, no adverse events, and no history of tracheal intubation or ventilator treatment. His mother was normal during pregnancy, and his growth and development were also normal. However, he appeared to have stridor, weak crying, and hoarseness at birth. The family doctor diagnosed congenital laryngomalacia and ordered vitamin D and calcium supplements. The symptoms improved but never completely disappeared. After admission, chest X-ray examination revealed bronchopneumonia. After treatment with glucocorticoid and antibiotics, the boy breathed smoothly, there was no cough, hoarseness decreased, and the moist rales gradually dissipated, but inspiratory stridor always existed, especially after activities or crying. Because of considering congenital diseases or space-occupying diseases of throat, we performed neck CT examination, which indicated linear diaphragm in glottis (Figure 1A). Laryngoscopy revealed that the anterior commissure of glottis was covered by webbed connection, which blocked nearly two thirds of glottis (Figure 1B). Laryngeal web separation was recommended, but his mother refused. After 1 month, the boy suffered from respiratory tract infection again, which caused difficulty in breathing, and could hardly make a sound. He was rushed to the emergency department. After giving symptomatic treatment, the child’s clinical symptoms were stable, and his mother agreed to the application for surgery. We chose the treatment of carbon dioxide (CO_2_) laser and cryotherapy by bronchoscope. During the operation, it could be seen that the laryngeal web covered the front joint of glottis, CO_2_ laser was given to gradually destroy the web along the midline of the laryngeal web to both sides, and the necrotic material was removed by forceps several times, and then the edge was frozen and trimmed. No other congenital bronchial malformations were found. Adrenaline injection (1:10,000, 1 ml) and budesonide suspension (1 mg) were injected through bronchoscope to reduce larynx edema. Dexamethasone (5 mg) was given to reduce larynx edema for three consecutive days. After the operation, the symptoms of stridor and hoarseness were significantly reduced. The second cryotherapy by bronchoscope was performed on the 10th day after operation. During the operation, a small amount of yellow necrotic material were found attached to the anterior commissure of glottis, and there was no adhesion of bilateral vocal cords. The mucosal smoothness was improved after cryotherapy (Figure 1C). On the third day after operation, the boy had no stridor, dyspnea, and hoarseness, and the timbre basically returned to normal, so he was discharged. One month, half a year, and 1 year after discharge, his pronunciation was completely normal, he had no dyspnea or stridor, and growth development was well; therefore, his guardian refused to ask for bronchoscopy again. We could not get the information by the bronchoscopy, but according to his clinical manifestation, he recovered very well.

## Discussion

Stridor is a harsh, vibratory sound of variable pitch caused by partial obstruction of the respiratory passages that results in turbulent airflow through the airway (1). Hoarseness is caused by the closure or vibration of vocal cords that is affected by various factors, resulting in air leakage or resonance obstacle, and bringing about timbre low or weak. Stridor and hoarseness are important symptoms of upper airway obstruction. Severe obstruction can lead to inspiratory dyspnea, cyanosis, and asphyxia, or can even be life-threatening. It is a respiratory emergency. After receiving such patients, it is most important for clinicians to quickly evaluate and judge the cause and degree of obstruction, and then relieve airway obstruction. The common causes of these symptoms include congenital dysplasia, infection, space-occupying disease, foreign body, trauma, and so on. Acute infectious cause is the most common among children presenting to emergency department with upper airway obstruction (1). However, in infants, congenital dysplasia should be given great attention, especially when family members can provide their infant’s history of stridor and hoarseness after birth; if the treatment effect of glucocorticoid and antibiotics is not satisfactory or can only be temporarily relieved, uncommon congenital factors should be considered, for example, the congenital laryngeal web mentioned in this paper. If the clinician thinks of the possibility of laryngeal web, it is not difficult to make a definite diagnosis through neck CT examination and laryngoscopy. However, it should be noted that infection, crying, and other factors may cause infants to be at risk of suffocation at any time. First aid facilities should be prepared. Because it may be difficult to endotracheal intubation, it is necessary to prepare for tracheotomy.

Congenital laryngeal web is caused by abnormal laryngeal development in the embryonic stage, representing less than 5% of congenital laryngeal anomalies (2). The formation of the larynx has experienced the process from the closure of the laryngeal cavity caused by laryngeal epithelial hyperplasia and fusion to the dissolution and absorption of the closed epithelium, then the re-establishment of the laryngeal cavity. If the dissolution and absorption process is blocked, a layer of fibrous connective tissue covered with squamous epidermis will be left in the larynx, which is called laryngeal webs. It was first reported in 1882 by Fleischmann in an autopsy study of an infant (3). Its pathogenesis may be related to 22q11.2 chromosome deletion (4, 5). The laryngeal web is divided into supraglottic web, interglottic web, and subglottic web according to the location of occurrence, which is more common in the interglottic type (6). It can cause hoarseness, stridor, laryngeal obstruction, and even serious dyspnea (7). Cohen classified congenital laryngeal web as follows based on clinical and anatomical considerations in 1985 (8). Type I is an anterior web involving 35% or less of the glottis. It is usually of uniform thickness, with little or no subglottic extension or stenosis; the voice is usually only slightly abnormal, with some hoarseness. Type II is an anterior web involving 35–50% of the glottis; there is little or no airway obstruction. The voice is husky and sometimes weak. Type III is an anterior web involving 50–75% of the glottis. Marked vocal dysfunction is present, with the voice being very weak and whispery. Airway obstruction is moderately severe, and an artificial airway is frequently necessary. Type IV is the most severe of all glottic webs, involving 75–90% or more of the glottis. The voice is aphonic and severe airway obstruction demands tracheotomy soon after birth.

It can be seen that stridor, hoarseness, and dyspnea depend on the location of the laryngeal web and the degree of glottic involvement. Although most severe congenital laryngeal web may be diagnosed shortly after birth, we cannot ignore those children whose symptoms are not obvious. They may only have mild symptoms for a period of time after birth, but in the process of growing up, because of some inducement, acute respiratory distress will occur (9). So we should improve the examination, make a clear diagnosis, and give corresponding treatment after actively saving lives.

The treatment strategy after diagnosis should be determined according to the classification of laryngeal web and the severity of children’s symptoms, and the acceptance of parents to the risk of surgery should also be considered. Parents usually cannot accept any surgical treatment when the child’s state is within the acceptable range, for example, when the boy was first hospitalized. The small anterior web sometimes does not need treatment or postpones pre-school treatment (10). For type I and II laryngeal webs, CO_2_ laser, scissor cutting, and balloon dilatation can be selected (11). For type III and IV laryngeal webs, tracheotomy and open surgery are preferred due to the presence of cartilaginous stenosis. At present, the simple separation of laryngeal web is mostly used in the treatment of patients with simple interglottic web (12). The method of separation is usually separated from the free edge along the midline of web, and attention should be paid to protecting the anterior interglottic tendon to avoid new injuries. However, this leaves two opposing raw surfaces that can predispose to re-formation of the web. We can put silicone keel or use mucosal flaps to maintain separation between healing tissues, preventing further scarring and recurrent web formation (13). In this case, the laryngeal web area was large, but there was no other airway malformation. Therefore, we chose CO_2_ laser and cryotherapy by bronchoscope. Through close observation after operation, the boy recovered well, so we did not give further treatment.

## Conclusion

Congenital laryngeal web is a rare but challenging laryngeal lesion. It is very important that the disease can be considered and examined for infants with recurrent stridor and persistent hoarseness after birth. Age, medical history, and response to treatment are very important for the diagnosis of laryngeal web. Combined with neck CT and laryngoscopy, it can be detected and diagnosed. The treatment strategy after diagnosis should be determined according to the classification of laryngeal web and the severity of children’s symptoms, and the acceptance of parents to the risk of surgery should also be considered.

## Data Availability Statement

The raw data supporting the conclusions of this article will be made available by the authors, without undue reservation.

## Ethics Statement

Ethical review and approval was not required for the study on human participants in accordance with the local legislation and institutional requirements. Written informed consent to participate in this study was provided by the participants’ legal guardian/next of kin.

## Author Contributions

The author confirms being the sole contributor of this work and has approved it for publication.

## Conflict of Interest

The author declares that the research was conducted in the absence of any commercial or financial relationships that could be construed as a potential conflict of interest.

## Publisher’s Note

All claims expressed in this article are solely those of the authors and do not necessarily represent those of their affiliated organizations, or those of the publisher, the editors and the reviewers. Any product that may be evaluated in this article, or claim that may be made by its manufacturer, is not guaranteed or endorsed by the publisher.
